# The Preparation and Characterization of Quantum Dots in Polysaccharide Carriers (Starch/Chitosan) as Elements of Smart Packaging and Their Impact on the Growth of Microorganisms in Food

**DOI:** 10.3390/ma14247732

**Published:** 2021-12-15

**Authors:** Wiktoria Grzebieniarz, Nikola Nowak, Gohar Khachatryan, Marcel Krzan, Magdalena Krystyjan, Jarosław Kosiński, Karen Khachatryan

**Affiliations:** 1 Faculty of Food Technology, University of Agriculture in Krakow, Balicka Str. 122, 30-149 Krakow, Poland; wiktoria.grzebieniarz@urk.edu.pl (W.G.); nikola.nowak@urk.edu.pl (N.N.); magdalena.krystyjan@urk.edu.pl (M.K.); jarek.kosinski.39@gmail.com (J.K.); karen.khachatryan@urk.edu.pl (K.K.); 2 Jerzy Haber Institute of Catalysis and Surface Chemistry, Polish Academy of Sciences, Niezapominajek Str. 8, 30-239 Krakow, Poland

**Keywords:** starch, chitosan, quantum dots, smart packaging

## Abstract

Nanocomposite materials are increasingly commonly used to ensure food safety and quality. Thanks to their unique properties, stemming from the presence of nanoparticles, they are used to develop advanced sensors and biosensors, e.g., for various harmful substances, heavy metals, microorganism growth, and environmental changes in food products. The aim of this study is to produce novel films based on natural resources—potato starch and chitosan—incorporating generated quantum dots of zinc sulfide and cadmium sulfide. The biocomposites were subjected to the following assays: FTIR spectroscopy, UV-VIS spectroscopy, photoluminescence spectroscopy, and SEM/TEM spectroscopy. Their mechanical properties were also analyzed, a colorimetric analysis was performed, and the water content, solubility, and water absorption capacity were determined. A storage test was also performed, using poultry meat covered with the produced films, to assess the microbiological quality. The results confirmed the presence of the quantum dots in the starch–chitosan matrix. The unique optical properties of the films were also demonstrated. It was shown that the composites with nanoparticles limited the growth of selected microorganisms in poultry meat. The food storage time was found to have an impact on the fluorescent properties of the composites. The results point to the possibility of using the produced films as active and smart packaging.

## 1. Introduction

In every aspect of life, we see a strong emphasis being placed on the environmental issues all around the world. Novel, recyclable materials are frequently approved for mass production. Meanwhile, films and packaging, made mainly of plastic, are used in practically every area of life.

For several years, research has been conducted on creating films that, thanks to their unique properties (biological, physical, and chemical), find use in medicine, agriculture, pharmacy, tissue engineering, biotechnology, and as anti-microbial agents [1,2,3]. Such an interest in these materials stems mainly from their content of bio-derivative or biodegradable polymers, which are a cheap and commonly available group of compounds. Depending on their origin, polysaccharides show anti-oxidant, immunomodulating, anti-inflammatory, antiviral, antimutagenic, carcinostatic, and anti-clotting properties [4,5,6]. This enables them to be used as an alternative to plastics, which carries numerous benefits for the environment.

Starch and chitosan are among the most common polymers used to produce sustainable food packaging materials. Chitosan is considered a biocompatible material, and its proven antimicrobial effect makes it suitable for use in medicine, tissue engineering, therapy, and pharmacy—for drug delivery.

The use of biocomposite films, developed to date for commercial purposes, is limited due to natural polymers’ poor barrier and mechanical properties. It appears, however, that the production of nanocomposites composed of multiple polymers can help to achieve interesting results [7]. Introducing a nanometric-scale component into a composite, results in a synergistic effect and, consequently, a material is produced with properties different from the properties of its individual components. This way, adhesion is improved, water absorption capacity is reduced, and the stability and resistance to environmental factors are increased [8,9].

Lately, a significant trend in nanotechnology has been a synthesis of polysaccharide composites, which serve as sensors of harmful substances, heavy metals, microorganism growth, and environmental changes [10,11,12]. A sensory nature can be produced in films by modifying them with quantum dots (QDs) with unique chemical, optical, physical, and electronic properties [13,14,15]. Due to their ability to fluoresce and their photostability, they have found use in detecting and reducing food pathogens, carcinogens in food products, and pesticides on the surface of fruits and vegetables [16,17,18,19,20]. With the use of nanomaterials, visible progress has taken place in the detection systems designed for monitoring gas releases, humidity and temperature changes, and microorganism growth in packaged foods. As a result, it is now possible to provide information on product quality and freshness based on the level, or changes in the level, of oxygen, carbon dioxide, pH, moisture, and specific chemical substances released during food spoilage [21,22,23]. This, in turn, has led to a significant improvement in food stability and color, as well as in the permeability, barrier, antibacterial, mechanical, and thermal properties. In medicine, nanosensors have enabled targeted drug delivery [24].

While substantial progress has been made, there is still a need for demonstrating biosensor functionality under realistic conditions. These goals include detection system streamlining (skipping stages or reagents), which is intended to reduce production costs and issues related to safety regulation compliance due to the potential migration of nanoparticles and their contact with food. Despite these limitations, this area shows promising potential [25]. To this end, a potato starch- and chitosan-based bionanocomposite was synthesized with generated ZnS and CdS semiconductor particles. The polymers and QDs were combined with the intent to see how such a configuration affects the mechanical, surface, and storage properties, color, and permeability. To this end, tests of the physiochemical, functional, and microbiological properties of the produced composites were conducted, such as: the colorimetric determination of film surface color, wetting angles, water content, mechanical properties, solubility, degree of swelling, SEM/TEM, DLS, photoluminescence spectroscopy, FTIR, and UV-VIS.

## 2. Materials and Methods

### 2.1. Materials

The following chemical reagents were used to produce the nanocomposites: potato starch (amylose: amylopectin ratio = 26:74, 12% moisture, Sigma-Aldrich, Poznan, Poland); chitosan (high molecular weight: 310,000–375,000 Da, degree of deacetylation > 75%, from shrimp shells, Sigma-Aldrich); acetic acid (99.5%, Sigma-Aldrich); glycerin (99.5%, Sigma-Aldrich); zinc acetate (99.999% trace metals basis, Sigma-Aldrich); ammonium sulfide (20 wt. % in H_2_O Sigma-Aldrich); and cadmium acetate (≥99.99% trace metals basis, Sigma-Aldrich).

For microbiological tests, the ALOA, TBX, PCA, PRI/RPF, MCCD, and VRBG (Biomaxima, Lublin, Poland) media were used.

### 2.2. Methods

#### 2.2.1. Polymer Matrix Preparation

The polymer matrix was obtained according to the procedure described in our previous paper [1,7]. A total of 800 g of 4% potato starch paste was prepared by weighing 32 g of starch in a beaker on a laboratory balance and supplementing it with 752 g of deionized water. The resulting solution was heated to 70 °C and stirred with a magnetic stirrer until the complete gelatinization of the starch was achieved. At the end of the process, 16 g of glycerin was added.

A total of 400 g of 2% chitosan solution was prepared by weighing 8 g of chitosan on a laboratory balance and supplementing it with 388 g of acetic acid solution (0.5%). The resulting samples were heated at 70 °C and stirred with a magnetic stirrer until the chitosan was completely dissolved. At the end of the process, 4 g of glycerin was added.

The produced potato starch and chitosan solutions were mixed together at a 2:1 weight ratio.

#### 2.2.2. Control Sample K Preparation

A total of 400 g of potato starch/chitosan mixture was placed in an ice bath with continuous stirring on a mechanical stirrer (Heidolph RZR 2020, Schwabach, Germany). After cooling the intensively stirred solution down to 4 °C, 24 cm^3^ of cooled deionized water was added dropwise and thoroughly mixed with the magnetic stirrer. After adding the water, the mixture was left for 15 min with even more intensive stirring. Then 100 g portions were transferred to Petri dishes with a 12 cm diameter, and left at room temperature until dry to create films.

#### 2.2.3. QD Sample Preparation—ZnS

Quantum dots were generated according to a procedure developed in our previous manuscripts [1,26,27,28]. A total of 400 g of potato starch/chitosan mixture was placed in an ice bath with continuous stirring on a mechanical stirrer (Heidolph RZR 2020). After cooling the intensively stirred solution down to 4 °C, 12 cm^3^ of cooled 0.1 M zinc acetate solution was slowly added dropwise. In the following stage, 12 cm^3^ of cooled 0.1 M ammonium sulfide solution was added dropwise. After adding the solutions, the mixture was left for 15 min with even more intensive stirring. Then 100 g portions were transferred to Petri dishes with a 12 cm diameter, and left at room temperature until dry to create films.

#### 2.2.4. QD Sample Preparation—CdS

A total of 400 g of potato starch/chitosan mixture was placed in an ice bath with continuous stirring on a mechanical stirrer (Heidolph RZR 2020). After cooling the intensively stirred solution down to 4 °C, 12 cm^3^ of cooled 0.1 M cadmium acetate solution was slowly added dropwise. In the following stage, 12 cm^3^ of cooled 0.1 M ammonium sulfide solution was added dropwise. After adding the solutions, the mixture was left for 15 min with even more intensive stirring. Then 100 g portions were transferred to Petri dishes with a 12 cm diameter and left at room temperature until dry to create films.

### 2.3. SEM/TEM Microscopy

The analyses of the sizes and morphologies of the as-prepared nanoparticles were studied using a high resolution JEOL 7550 scanning electron microscope equipped with a TEM detector. Samples were prepared after drop-coating 10 µL of the sample on carbon coated grids 200 mesh Cu (100) (TAAB Laboratories, Aldermaston, Berks, UK).

### 2.4. FTIR Spectroscopy

The FTIR spectra of the produced composites were analyzed within the 4000–700 cm^−1^ range, using a MATTSON 3000 FT-IR spectrophotometer (Madison, WI, USA) equipped with a 30SPEC 30 Degree Reflectance adapter (MIRacle ATR, PIKE Technologies Inc., Madison, WI, USA).

### 2.5. UV-VIS Spectroscopy

The UV-VIS absorption spectra of the bionanocomposites were analyzed using a Shimadzu 2101 scanning spectrophotometer (Shimadzu, Kyoto, Japan) within the 200–700 nm range.

### 2.6. Surface Color Measurements

The surface color was measured using Konica MINOLTA CM-3500d equipment (Konica Minolta Inc., Tokyo, Japan), with a 10 mm diameter window, using a reference D65 illuminant/10° observer. The results were expressed using the CIELab system. The parameters, such as L* (L* = 0 black, L* = 100 white); a*—share of the green color (a* < 0) or red (a* > 0); and b*—share of blue (b* < 0) or yellow (b* > 0), were determined on a white background standard and carried out on the day of preparing the dry foils. The experiment was repeated 5 times.

### 2.7. Mechanical Properties of the Films

Based on the procedure performed by Krystyjan et al. (2017), the dry foils were conditioned in desiccators at 25 °C and 52% relative humidity (RH), using saturated magnesium nitrate-6-hydrate for 48 h prior to analysis. The thickness of the obtained foils was measured with a micrometer (Sylvac SA, Crissier, Switzerland, catalog no. 805.1301), using a 0.001 mm resolution. The sample thickness was measured in five repetitions in various places.

The samples for the textural analysis were prepared according to ISO standards [ISO 527-1:2019] and determined using a TA-XT plus texture analyzer (Stable Micro Systems, Haslemere, UK). The films were cut into 35 × 6 mm strips and put into holders. The initial grip separation between the holders was 20 mm and the rate of grip separation was 2 mm/min. The tensile strength (TS) was calculated by dividing the tensile force (maximum force at rupture of the film) by the cross-section area of the film. The percentage of elongation at the break (EAB) was calculated by dividing the elongation at rupture by the initial gauge length and multiplying by 100. The average values of 10 replications were presented in the results.

### 2.8. Water Content, Solubility, and Degree of Swelling Determination

A total of 2 × 2 cm squares were cut out of the K, QD-ZnS, and QD-CdS samples, and weighed on an analytical balance (*m*_1_). The samples were then dried in a dryer at 70 °C for 24 h and reweighed (*m*_2_). The squares were then placed in beakers containing 30 mL of deionized water, covered, and stored for 24 h at room temperature (22 ± 2 °C). The remaining water was discarded, and the samples were dried on their surface with filter paper, then weighed (*m*_3_). The sample residues were then dried in a dryer at 70 °C for 24 h and subsequently weighed (*m*_4_). For each sample, three measurements were made and the average parameter value was assigned [29]. The values were then calculated using the following formulas:

(1) $$\mathrm{Water}\;\mathrm{content}\;[\%]=\frac{\left(m_{1}-m_{2}\right)}{m_{1}}\times 100$$

(2) $$\mathrm{Solubility}\;[\%]=\frac{\left(m_{2}-m_{4}\right)}{m_{2}}\times 100$$

(3) $$\mathrm{Degree}\;\mathrm{of}\;\mathrm{swelling}\;[\%]=\frac{\left(m_{3}-m_{4}\right)}{m_{3}}\times 100$$

The equation for calculating the degree of swelling was modified by including the dissolved portion of the film, so the end dry weight (*m*_4_) was considered for the calculations.

### 2.9. Contact Angle Determination

The contact angles were determined using a Kruss-DSA100M (Kruss GmbH, Hamburg, Germany). The sessile drop method was used to determine the distilled water and pure diiodomethane contact angles on the studied polysaccharide foil surfaces. The detailed methodology of the contact angle experiments and surface free energy analyses was presented in our previous paper [A]. We used the Owens–Wendt method [B], which is the best for polymer property evaluation [C]. All measurements were performed in an environmental cell, under constant temperature conditions (22 ± 0.3 °C) and humidity. For each foil sample, a minimum of four successive tests were carried out.

### 2.10. Particle/Aggregate Sizes (DLS) and Zeta Potential

The zeta potential and particle/aggregate sizes were measured using the Malvern Zetasizer Nano ZS apparatus with disposable measurement cells (DTS 1065, Malvern). The zeta potential was calculated from the electrophoretic mobility of particles using the Smoluchowski model. The results are expressed as an average from the measurements of 20 consecutive runs. All measurements were performed in the water mixtures obtained after the dissolution of the developed package film in 1%wt. acetic acid (this method was used to dissolve chitosan fully). The foil samples with 0.1g (±0.01 g) were dissolved in 5 mL of 1%wt. of water solution of acetic acid. A magnetic stirrer stirred the mixtures for 1 h until the full dissolution of foil film.

### 2.11. Microbiological Testing

Wheaton™ 100 mL sterile polystyrene universal containers were prepared, two repetitions for each of the films generated (K, QD-CdS, and QD-ZnS), and additionally for the polyethylene stretch foil for food packaging (FS). In each container, 5 poultry meat samples were placed, each weighing 1 g (±0.05 g), then a piece of 1 of the films was placed in the cap, and the containers were closed and moved to a cold storage. The material was stored for 3 days, and a sample (1 g) was taken from each container every day. Microbiological assays were performed in accordance with the PN-EN ISO 4833-2:2013-12+AC:2014-04, PN-EN ISO 21528-2:2017-08, PN-EN ISO 6888-2:2001+A1:2004, PN-EN ISO 11290-1:2017-07, PN-EN ISO 10272-2:2017-10, and PN-ISO 16649-2:2004 standards.

The results of the microbiological tests were analyzed and presented for each microorganism separately. The statistical analysis included the comparison of each tested film sample separately for the times of 24, 48, and 72 h. The Tukey’s test and one-way analysis of variance were used to perform the statistical analysis.

### 2.12. Photoluminescence Spectroscopy

The photoluminescence measurements of the films were performed at room temperature using a F7000 HITACHI spectrophotometer. The wavelength of 360 nm was used for the excitation. The emission spectra of the films were measured before the storage test and after five days of storing the poultry meat under the films.

## 3. Results and Discussion

### 3.1. Surface Color of Foils

The color parameters of the foils are summarized in Table 1. It was observed that the L* parameter ranged from 94.30 to 95.55, suggesting that the foils were considered light. The generated CdS quantum dots in the starch/chitosan foils did not affect the brightness of the sample. The synthesized ZnS quantum dots in the same polysaccharide matrix contributed to the lightening of the obtained foils. The QD-CdS samples had a higher proportion of green (a* < 0) and yellow (b* > 0) colors than the control and QD-ZnS samples (Table 1). This view is the result of absorbing light with a different wavelength than the K and QD-ZnS samples, which was confirmed by the measurements of the UV-VIS spectrum. For the K and QD-ZnS, the differences between the a* and b* parameters was not statistically significant (Table 1) as they absorbed UV light radiation to a similar extent.

### 3.2. Mechanical Properties of the Foils

The thickness, tensile strength (TS), and percent elongation at break (E) of the starch- and chitosan-based biocomposites are shown in Table 2. The thickness of the films obtained from the bionanocomposites differed from each other and varied, from 0.118 mm to 0.133 mm, even though the same amount of solution was poured into the trays. The thickness of the control sample was 11.8–12.7% less than the QD-ZnS and QD-CdS. In the opinion of Krystyjan et al. [7], this resulted from the enrichment of the solids content in the obtained foils (QD-ZnS and QD-CdS). The addition of the ZnS and CdS quantum dots to the starch/chitosan foils reduced their tensile strength and elongation at break. These changes can result from the significant increase in the size of the aggregates in the foils, which weakened the structure of the system. As the DLS measurements confirmed, the resulting aggregates were characterized by various sizes, from 510 to 3600 nm, while the control samples without quantum dots were made of colloidal particles with a diameter of 350 nm as we confirm later in this work. Our previous research shows a decrease in the molecular weight of potato starch polysaccharide chains after the generation of QDs in its solution, which, in effect, reduced the viscosity of the gels [27,30] and, thereby, contributed to the weakening of the mechanical properties of the foils.

### 3.3. Determination of Water Content, Solubility, and Degree of Swelling

The water content, solubility, and degree of film swelling are shown in Table 3.

All the films have a similar, low water content. It can be observed that the film with the ZnS quantum dots shows better solubility than both the film with the CdS quantum dots and the control film, which are soluble to a similar degree. Each film shows an almost identical degree of swelling.

Generating the quantum dots, both ZnS and CdS, in the starch/chitosan polysaccharide matrix did not significantly impact the water content or the degree of swelling of the produced films. The presence of the ZnS quantum dots increased the solubility of the produced film.

### 3.4. Optical Properties

**Figure 1:**
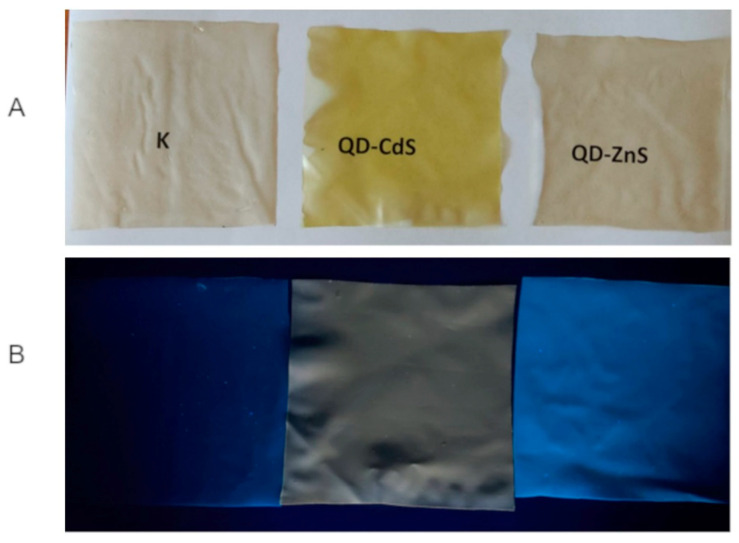
Starch–chitosan films: (**A**)—in daylight, and (**B**)—in the dark excited with the UV (365 nm) radiation.

The produced biopolymer films were subjected to a simple optical test. As a result of exposing the films to UV radiation using a lamp, fluorescence was observed in all three samples (Figure 1). The control sample showed blue fluorescence, resulting from the emission properties of chitosan [31]. The QD-ZnS sample showed a dark blue/violet fluorescence. The composite with the cadmium sulfide quantum dots showed an orange fluorescence. The different colors of the light emitted by the produced films are the first proof of the presence of the quantum dots in the starch–chitosan matrix, and the differences in the color of the emitted light indicate that the quantum dots produced differed in size [32].

### 3.5. SEM/TEM Microscopy

**Figure 2:**
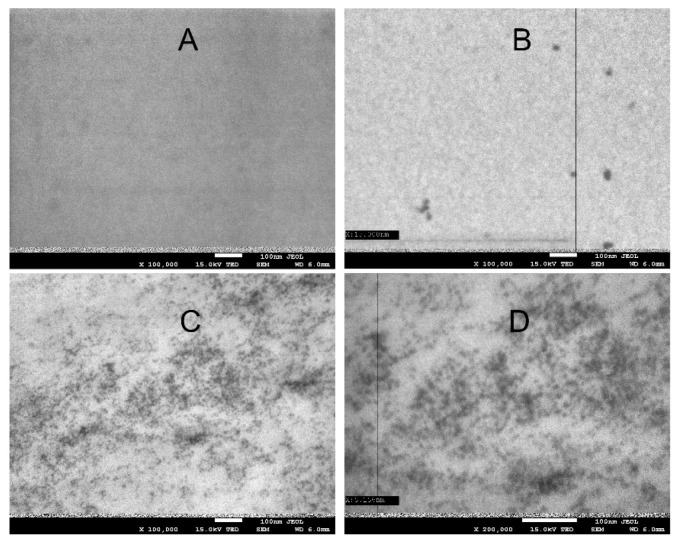
SEM/TEM image of the biocomposites: (**A**)—control, (**B**)—QD-CdS, and (**C**,**D**)—QD-ZnS, at ×100,000, ×200,000 zoom, respectively.

Figure 2 shows images of the starch–chitosan films: control, with ZnS quantum dots, and CdS dots, made using a scanning electron microscope with a detector for TEM measurements. No presence of quantum dots was observed in the control film. The QD-ZnS film contained quantum dots sized about 3–5 nm, while the QD-CdS film had dots sized about 10–20 nm. The larger size of the CdS dots is most likely caused by the size of the cadmium atom, which is much larger than the zinc atom.

### 3.6. FTIR Infra-Red Spectra

**Figure 3:**
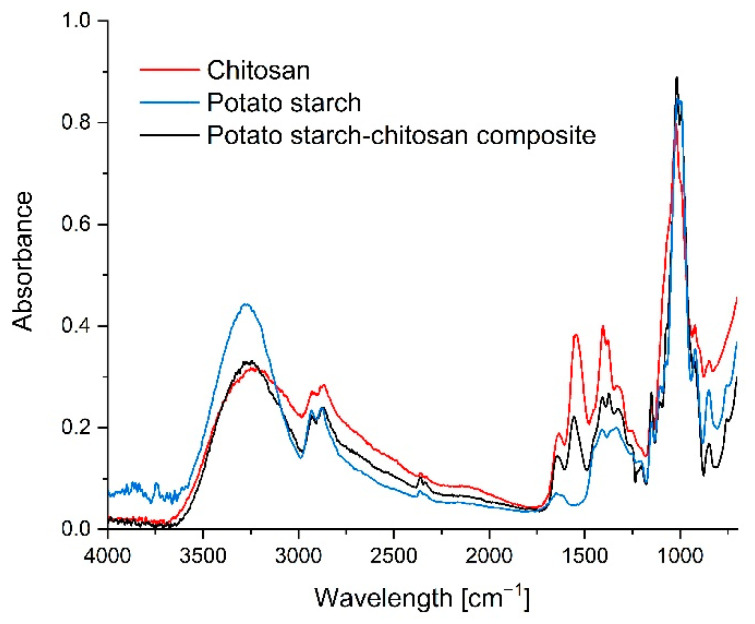
FTIR spectra of chitosan, potato starch, and the starch–chitosan composite.

**Figure 4:**
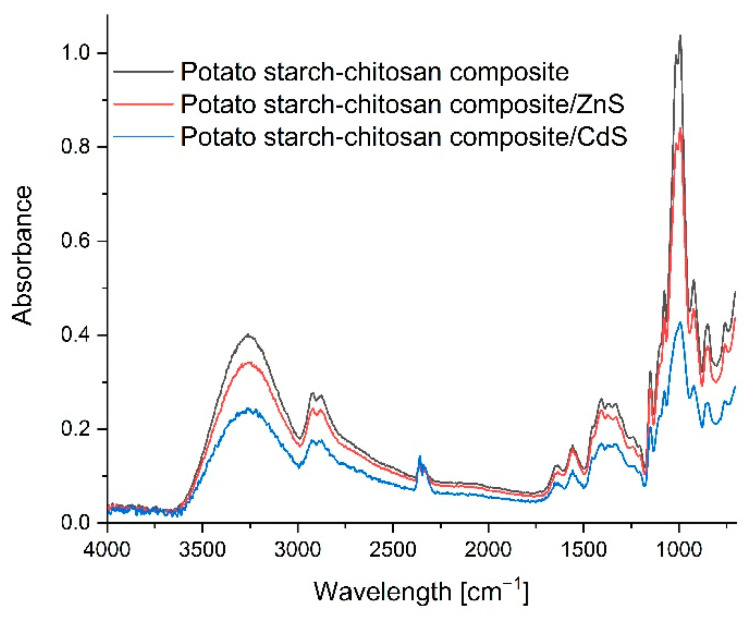
FTIR spectra of the control film, ZnS quantum dot film, and CdS quantum dot film.

The FTIR spectroscopy testing was performed to investigate the interactions between the chitosan and starch (Figure 3), and between the quantum dots and the chains of these polysaccharides (Figure 4).

The wide band present at 3251 cm^−1^ in the chitosan spectrum corresponds to the OH group stretching vibrations, and to the overlapping bands of the NH group stretching vibrations. The band at 1578 cm^−1^ corresponds to the vibrations of the NH groups (amide II). The band near 1655 cm^−1^ corresponds to the carbonyl group stretching vibrations (amide I). In the starch spectrum, the wide band at 3350 cm^−1^ corresponds to the OH group stretching vibrations. The multiple bands in the 1150–950 cm^−1^ range correspond to the asymmetric vibrations of the C–O–C bridge bonds (1150 cm^−1^), asymmetric vibrations of rings (about 1100 cm^−1^), and stretching vibrations of the (C–O) bond (1080–960 cm^−1^ range). Multiple bands are observed at 2916–2936, 2855, 1405–1465, and at 1245 cm^−1^ in the spectra of both samples (starch and chitosan), which come from the -CH_2_- group, and at 2880–2900 and 3200 cm^−1^, which correspond to the C-H units in the polysaccharide chains. The chitosan/starch composite film spectrum shows that the addition of starch caused the band corresponding to the chitosan amine group to shift from 1578 cm^−1^ to 1584 cm^−1^. This result points to the interactions between the hydroxyl groups of starch and amine groups of chitosan. The hydroxyl group band could not be used to assess the interactions, due to the masking effect of the added glycerin [7,33].

Figure 4 shows the spectra of the starch–chitosan film, ZnS quantum dot film, and CdS quantum dot film. Essentially, the control film and the quantum dot composite spectra are very similar, and no significant displacements of bands are observed. Only the differences in the intensity of absorbance of the individual composites can point to the forming of hydrogen bonds between the components, to different film thicknesses, and to different water contents, which is in agreement with the results obtained during the measurements of the thickness and water absorption capacity of the produced composites. Therefore, it can be concluded that generating quantum dots, both ZnS and CdS, does not impact the structure of the polysaccharides, which serve only as the matrix for the synthesized nanoparticles [34].

### 3.7. UV-VIS Spectroscopy

**Figure 5:**
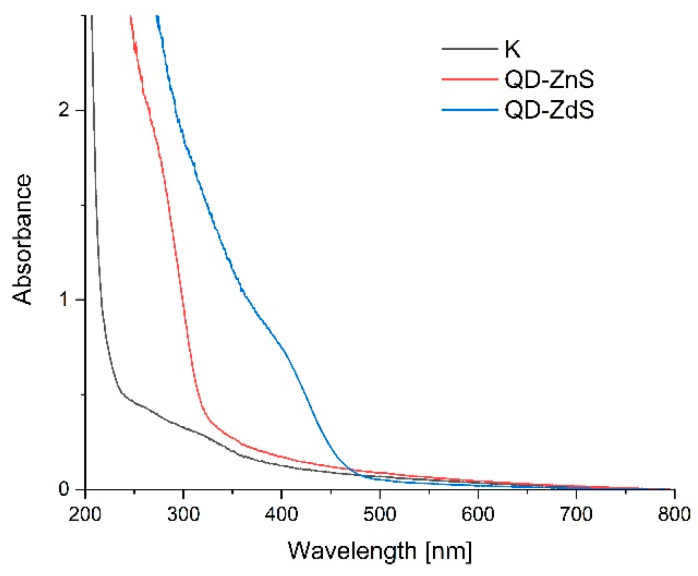
UV-VIS spectra of the control film (K), ZnS quantum dot film (QD-ZnS), and CdS quantum dot film (QD-CdS).

Figure 5 shows the UV-VIS absorption spectra of the produced films. The band at about the 250–350 nm wavelength indicates the absorption of radiation from the UV light range by the control sample. The nanocomposite with the ZnS quantum dots absorbs radiation approximately in the 280–350 nm wavelength range, which corresponds to visible violet and blue light, and CdS—380–480 nm, which corresponds to yellow light.

The results indicate the presence of quantum dots in the polysaccharide matrix. The widths of the bands is evidence of the different sizes of the synthesized nanoparticles, which has already been corroborated by the images from the scanning electron microscope (SEM). The band in the UV radiation range indicates that very small zinc sulfide dots were produced. The cadmium sulfide dots are slightly larger, which is confirmed by the shift of the band to higher wavelengths. A quantum dots size can be determined based on their position—the relation between the nanoparticles’ size and their UV-VIS spectrum is commonly known in the literature [32,35]. The band present in the control sample in the 250–350 nm range is characteristic for chitosan [31].

### 3.8. Wetting Angles

The investigations of the water and diiodomethane wetting conditions showed that the studied starch–chitosan films are hydrophobic (Table 4). The least hydrophobic was the control film without the addition of quantum dots (water contact angle 69.7°), and the most hydrophobic was the film containing CdS quantum dots (88.0°). The analyses of the surface energies of water carried out, based on the obtained contact angles of water and diiodomethane (by the Owens–Wendt method), show that the hydrophobic properties of the tested materials are mainly due to dispersion forces. There was practically no influence of polar forces in the samples. The control foil without quantum dots was the most polar.

### 3.9. DLS

The analysis of the sizes and zeta potentials shows (Table 5) that the films are composed of aggregates of various sizes and surface properties (zeta potentials). The control foils without quantum dots are made of colloidal particles with a diameter of 350 nm. The addition of quantum dots causes a significant increase in the size of the aggregates. The particle size for CdS is 510 nm and for ZnS is 3600 nm. The zeta potential varies from 51.4 mV for the control foil, through 35.6 mV for the CdS foil, to −7.4 mV for the ZnS foil.

### 3.10. Storage Test and Microbiological Testing

The most common agents that cause diseases and infections due to the consumption of poultry meat are bacteria of the *Enterobacteriaceae* (*Salmonella*, *Escherichia coli*), *Campylobacter*, *Listeria*, and *Staphylococcus* genera. In raw poultry meat, the pathogenic bacteria count ranges from 1% to 10%, depending on external factors [36].

**Figure 6:**
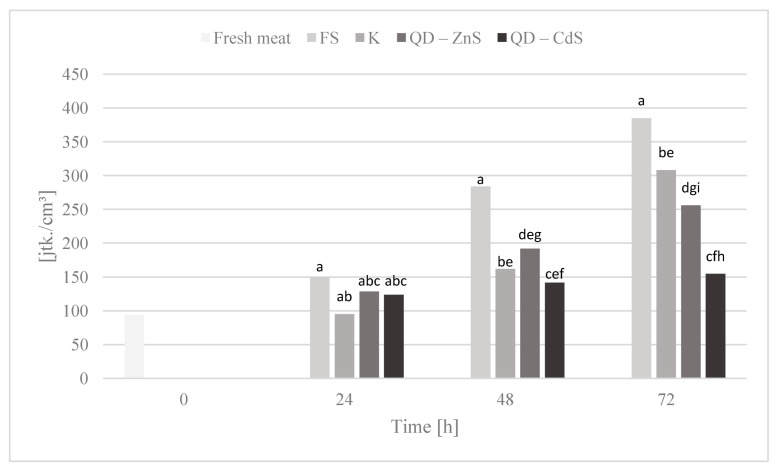
Total microbial count in poultry meat stored under different films at about 4 °C. The parameters in the columns denoted with the same letters (a, b, c, etc.) do not differ statistically at the confidence level of *p* < 0.05.

**Figure 7:**
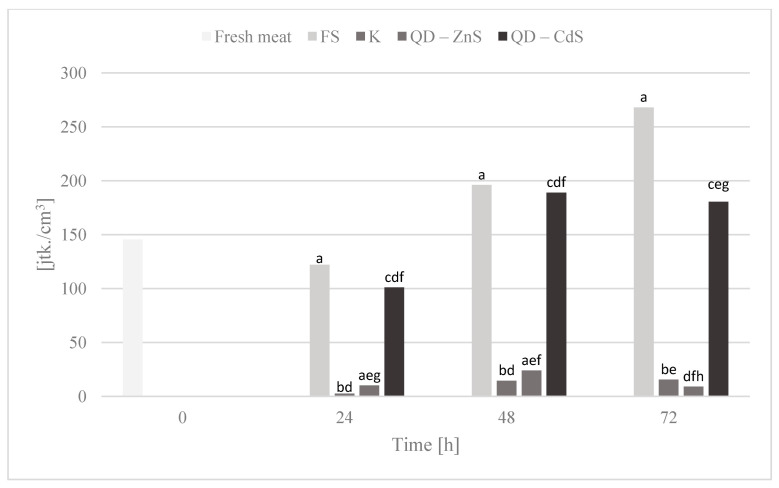
*Enterobacteriaceae* bacteria count in poultry meat stored under different films at about 4 °C. The parameters in the columns denoted with the same letters (a, b, c, etc.) do not differ statistically at the confidence level of *p* < 0.05.

**Figure 8:**
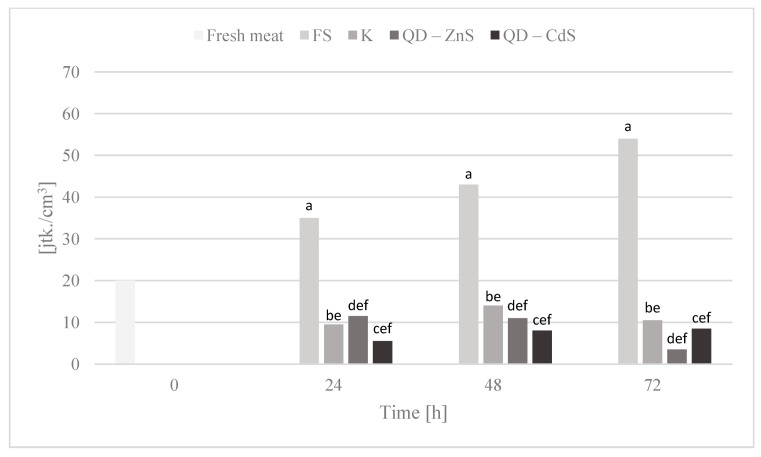
*Escherichia coli* bacteria count in poultry meat stored under different films at about 4 °C. The parameters in the columns denoted with the same letters (a, b, c, etc.) do not differ statistically at the confidence level of *p* < 0.05.

**Figure 9:**
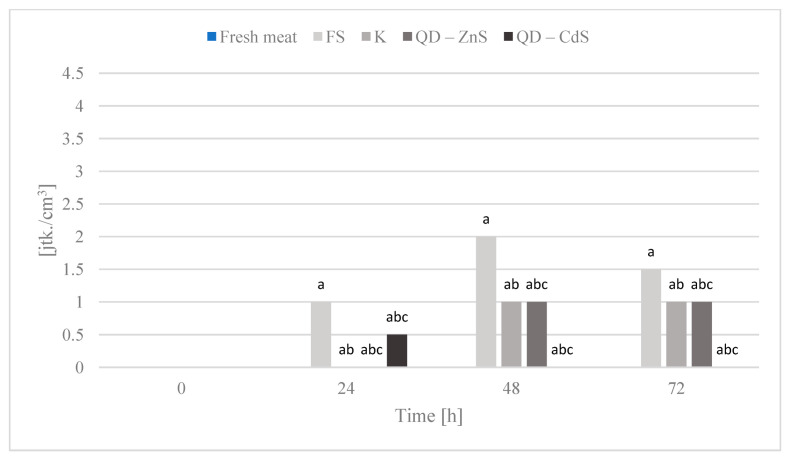
*Campylobacter* spp. Bacteria count in poultry meat stored under different films at about 4 °C. The parameters in the columns denoted with the same letters (a, b, c, etc.) do not differ statistically at the confidence level of *p* < 0.05.

**Figure 10:**
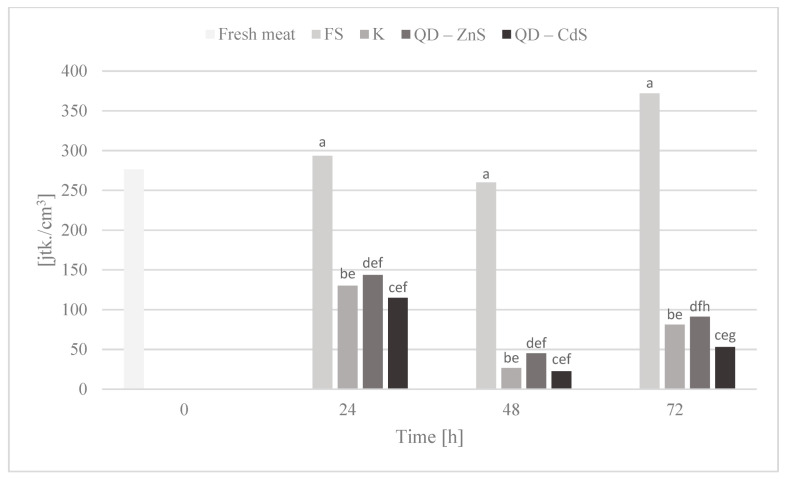
Coagulase-positive *Staphylococcus* count in poultry meat stored under different films at about 4 °C. The parameters in the columns denoted with the same letters (a, b, c, etc.) do not differ statistically at the confidence level of *p* < 0.05.

The purpose of the storage test was to assess the microbiological quality of poultry meat stored under different types of films at a cold storage temperature of about +4 °C. Figure 6, Figure 7, Figure 8, Figure 9 and Figure 10 show the results of the microbiological inoculations for individual groups and species of bacteria as a function of time.

An important quality of quantum dots is their antibacterial activity. When compared with the activity of antibiotics, they are characterized not only by their luminescence capability, but also by their high structural stability [37,38]. The modification with polymers reinforces their antimicrobial effect, while their dissimilarity from common antibiotic treatments enables them to combat microorganisms resistant to pharmaceuticals and allows pharmaceuticals to be gradually replaced, which, at a time of rising resistance, is very important [39,40,41]. The microbiological tests we performed confirm the above conclusions, as demonstrated in Figure 6, which shows that the total microorganism count changes in meat during poultry meat storage. It was observed that all biocomposite films noticeably limited the growth of microorganisms compared to polyethylene stretch foil for food packaging(FS). The exception to this is the analysis after 24 h, in which no significant differences were observed between the tested samples.

The highest growth inhibition was determined for the film with cadmium sulfide dots. Lower values were found only for the bacteria of the *Enterobacteriaceae* family (Figure 7), where a rapid halt to the growth of this bacteria group is observed for the control film and the film with the zinc sulfide quantum dots. Cadmium sulfide quantum dots have a weaker effect on the growth of these microorganisms, but, in time, a noticeable inhibition of microorganism growth is observed, unlike the food film. The growth of *Escherichia coli* bacteria was inhibited by all of the biocomposite films, in contrast to the food film (Figure 8). Galdiero et al. found that ZnS QDs were generally more active against Gram-negative bacteria than Gram-positive ones [42,43]. It was found that the bacteria of the *Campylobacter* spp. family are not sensitive to any of the films (Figure 9). It appears that the presence of quantum dots does not affect the antibacterial activity for this bacteria group, which was also confirmed by the performed statistical analysis. It was observed that the composite films noticeably inhibited the growth of coagulase-positive *Staphylococci*, compared to the food film (Figure 10). No presence of *Listeria monocytogenes* was detected in the test product, neither in the fresh meat nor after storage under each of the films (not shown in the chart). However, no definitive conclusions concerning the antimicrobial effect of the film on these bacteria can be drawn, as they were missing during the first three days of storing the meat.

The growth inhibition of the selected bacteria groups can be explained by the demonstrated toxic effect of the nanoparticles on bacteria cells, resulting from their high surface-to-volume ratio. The small size of the quantum dots enables them to penetrate cell walls and interact with functional biomolecules, such as proteins and DNA. Based on the experimental evidence, it was concluded that quantum dots have a strong antibacterial effect, which involves bacterial cell wall breaking, cytoplasm leakage, and damage to cell structures caused by oxidative stress and leads to lysis of bacteria cells. A strong effect inhibiting the growth of *Enterobacteriaceae* by zinc sulfide nanoparticles was also demonstrated. Based on the literature, it can be concluded that the antibacterial action of the nanoparticles depends, to a large degree, on their size. This explains the weaker or delayed antimicrobial effect on *Enterobacteriaceae* bacteria of the film with the cadmium sulfide quantum dots. Their larger size than the ZnS dots was demonstrated in earlier analyses [44,45].

Other studies note the importance of oxidative stress caused by reactive oxygen species, which accelerate the destruction of biomolecular structures. Reactive oxygen species (ROS) is a general term describing molecules and reactive intermediate products with a strong positive redox potential. It was demonstrated that different types of nanoparticles were able to generate reactive oxygen species by reducing oxygen molecules. The main factors that generate ROS are restructuring, defect sites, and oxygen holes in crystals. It was confirmed that oxidative stress is the key factor affecting permeability changes in cell membranes, which can damage the cell membranes in bacteria. Inducing oxidative stress is an important antibacterial mechanism of nanoparticles. Under normal conditions, the production and removal of ROS in bacteria cells is balanced. However, with excess ROS production, the redox balance of a cell favors oxidation. This unbalanced state causes oxidative stress, which damages the individual components of the bacteria cells and makes it impossible for them to breathe or replicate, leading to cell death. It is assumed that the microorganism growth inhibition in our study was caused mainly by the oxidative stress caused by the presence of the quantum dots. The test films did not touch the product directly, so the interaction of the nanoparticles with the bacterial cells was greatly hindered [38,39,45].

The impact of the presence of chitosan on the antibacterial properties and cell growth environment cannot be ignored, either. There are three mechanisms of chitosan’s antibacterial action: the ionic interactions on the cell surface, causing cell liquids to leak through the cell wall; the inhibition of mRNA and protein synthesis by chitosan permeation into microorganism cell nuclei, forming an external barrier, chelating metals; and reducing the absorption of nutrients necessary for microorganism growth. Most likely, all of these mechanisms take place at the same time, but with different intensities. Important factors affecting the antibacterial properties of chitosan are its molecular mass and degree of acetylation. The lower the molecular mass and degree of acetylation, the stronger the growth inhibition effect on microorganisms. It is not confirmed, however, whether chitosan has a higher activity against Gram-positive or Gram-negative bacteria. It appears to affect the two species differently, although satisfactorily in both cases [46]. Due to its high chemical stability and internal compatibility, as well as low dissolving capacity, we used chitosan in our study for surface biomodification. Its use caused a normalization or even elimination of the potential toxicity of Cd- or Zn-containing QDs [47,48]. In a study by S.M. de Carvalho et al. [49], it was demonstrated that, when modified with chitosan, Cd-containing quantum dots showed no toxicity on three cell lines in vitro when chitosan was used in low concentrations and the incubation time was short [49]. It should be noted that significant water leakage was observed in all samples during the storing of poultry meat. Water vapor condensation on the walls of the containers was also observed. Therefore, there is the possibility that it had direct contact with the biocomposites, leading to a reinforcement of the antibacterial effect. It is worth noting that the very low amount of the quantum dots and chitosan that may have potentially reached the product in the condensed water vapor was sufficient to sharply reduce microbe growth.

Despite the lack of direct contact between the bionanocomposites with the test meat, there are concerns about their potential toxicological effects on humans and ecosystems. Currently, there are many controversies around the use of quantum dots in biological applications due to their potential release, which determines their interaction, bioaccumulation, and transfer to the environment [50,51,52,53]. The literature data concerning the toxicity spectrum of quantum dots are varied and contradictory due to the differences in the physicochemical properties of different QD types (composition, size, surface charge, and functionalization), as well as the lack of toxicological studies, or the high variety of the concentrations used [54]. This issue was greatly systematized by a study by Eunekeu Oh et al. [55], who conducted a meta-analysis of over 300 papers concerning the effects of Cd QDs on living organisms, and identified 1741 variants of their use and effects [55]. It is established that Cd, in ionic form, has a higher toxicity for living organisms than Cd-based quantum dots [56]. A study by Matos B. et al. on the organisms of the freshwater fish *Danio rerio* exposed for 7 days to the effects of Cd and Zn QDs in a range of concentrations from 10 to 1000 µg/L, showed low or moderate toxicity of these compounds [57]. In a study by Ye L. et al. [58], no evidence of toxicity was found after injecting CdS and ZnS quantum dots. The analyses of blood and biochemical markers showed no changes, while histological examinations revealed no irregularities. Nevertheless, after 90 days from the injections, a portion of the applied Cd dose was found in the liver, spleen, and kidneys, which indicates that quantum dot decomposition and their removal from the body occurs relatively slowly [58].

### 3.11. Photoluminescence Spectroscopy

To confirm the optical properties of the produced films and any sensitivity to changes in the environment, the photoluminescence spectra were measured.

**Figure 11:**
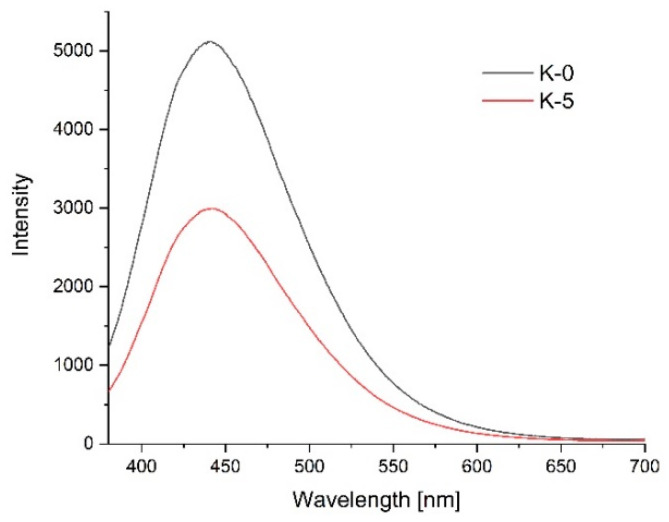
Emission lines of the control film before poultry meat storage (black line) and after 3 days of storage (red line).

**Figure 12:**
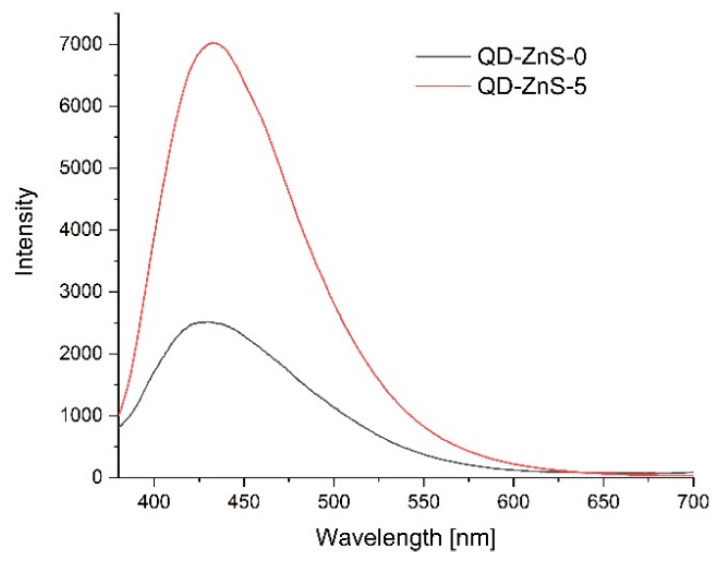
Emission lines of films with ZnS quantum dots before poultry meat storage (black line) and after 3 days of storage (red line).

**Figure 13:**
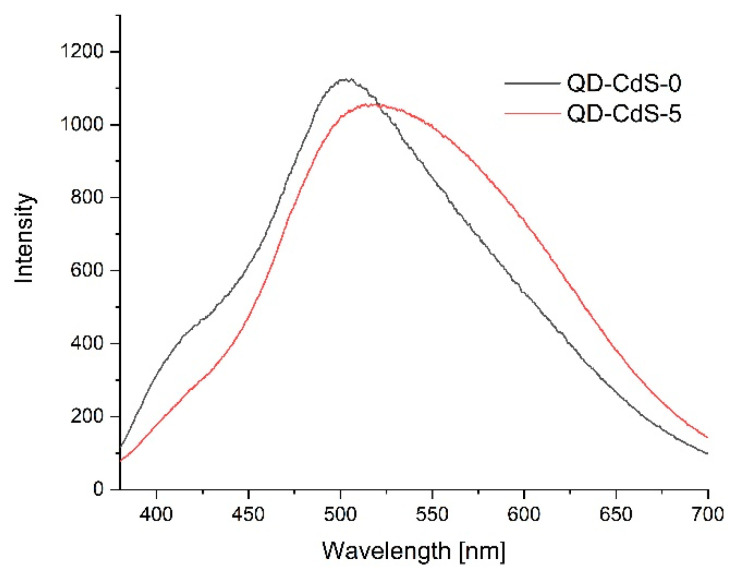
Emission lines of films with CdS quantum dots before poultry meat storage (black line) and after 3 days of storage (red line).

Figure 11, Figure 12 and Figure 13 show the emission spectra of the composites before conducting the storage test (black line) and after five days of storing poultry meat samples under the composites (red line).

Figure 11 shows the emission spectrum of the control film. The starch–chitosan composite showed significant light emissions at about the 440 nm wavelength. After three days of storing the meat underneath it, the emission intensity of this film was significantly reduced. The reduction in the control film intensity can result from a loosening of the chitosan structure due to absorbing water released from the meat.

Figure 12 shows the emission spectrum of the chitosan–starch film with zinc sulfide quantum dots. In the case of this nanocomposite, the light emissions at about the 430 nm wavelength, after storing the meat underneath it, sharply rose.

Figure 13 shows the emission spectra of the chitosan–starch film with cadmium sulfide quantum dots. In this case, after conducting the storage test, a slight reduction in emissions was observed, but a peak shift from about 500 nm to about 530 nm was noted.

The observed significant changes in the emissions of all films may have been caused by the sensitivity of these composites to changes occurring in the stored food. The products of biochemical and physicochemical transformations, which include ethanol, acetone, ethyl acetate, methyl benzoate, heptane, C15, C12, methyl-ethyl ketone, carbon disulfide, dimethyl sulfide, hexanal, and toluene, as well as free amino acids and water-soluble polypeptides, can interact with quantum dots, affecting the optical properties of the composites. Particular attention should be paid to the sulfur compounds and sulfur-containing amino acids released, as they can be doped on the quantum dot surface, directly affecting its emission properties [59].

## 4. Conclusions

The films based on natural polysaccharides—potato starch and chitosan—were successfully produced, and zinc sulfide and cadmium sulfide quantum dots were generated in them. The polysaccharides used do not interact significantly with each other, and generating the quantum dots did not cause structural changes in the polysaccharide chains. The biopolymers only formed a matrix and a carrier for the quantum dots generated. It was demonstrated that the quantum dots differed in size, and the films that contained them gained unique optical properties at minimal quantum dot concentrations. Tests also confirmed the fluorescent properties of chitosan and a change in emission intensity depending on the ambient conditions. Physicochemical analyses demonstrated that the films absorbed light radiation in a range characteristic for them, which opens the possibility for their potential use in protecting selected food products against the adverse effects of light radiation. An assessment of the mechanical properties of the composites showed that the presence of the quantum dots reduced the films’ rupture strength and tensile strength, compared to the control. Nevertheless, the films possess attractive properties, and further research on improving their mechanical qualities is advisable. It was demonstrated that the biocomposites are poorly soluble in water and are characterized by a significant water absorption capacity.

The storage test demonstrated that the produced films show bacteriostatic or bactericidal properties. The biocomposites greatly contributed to limiting the increase in the total microorganism count and the growth of *Escherichia coli* bacteria and coagulase-positive *Staphylococci* in poultry meat, likely contributing to extending the shelf life of this product. A difference in the antibacterial effects of the nanocomposites on *Enterobacteriaceae* bacteria was observed, depending on the quantum dot used. No antibacterial effect on *Campylobacter* ssp. bacteria was observed. In the case of *Listeria monocytogenes*, microbiological tests did not show its presence in the test meat during the five-day storage test.

With the results obtained, it can be concluded that the films produced can be an attractive element of packaging for selected products. The biocomposites meet the criteria of active and smart packaging. There are, however, rich possibilities for research in the area of novel packaging materials based on green chemistry and nanotechnology. The demonstrated indirect interaction of the quantum dots with microorganisms in food opens the possibility of developing novel biosensors forming an integral part of packages for the selective—both qualitative and quantitative—determination of microorganisms in food products.

## Figures and Tables

**Table 1:** Color parameters of the foils.

Sample	L* (D65)	a* (D65)	b* (D65)
K	94.6 ± 0.4 ^b^	−0.7 ± 0.1 ^b^	7.2 ± 0.8 ^b^
QD-ZnS	95.6 ± 0.1 ^a^	−0.8 ± 0.04 ^b^	7.6 ± 0.3 ^b^
QD-CdS	94.3 ± 0.04 ^b^	−11.4 ± 0.07 ^a^	45.0 ± 0.6 ^a^

The measurement was performed in 5 repetitions. The parameters in columns (value ± standard deviation) denoted with the same letters (a, b) do not differ statistically at the confidence level of *p* < 0.05.

**Table 2:** Mechanical properties of the foils.

Sample	Thickness (mm)	TS (MPa)	E (%)
K	0.12 ± 0.005 ^b^	7.3 ± 0.6 ^a^	59± 5.0 ^a^
QD-ZnS	0.13 ± 0.005 ^a^	5.7 ± 0.5 ^b^	47.1 ± 6.2 ^b^
QD-CdS	0.13 ± 0.001 ^a^	4.6 ± 0.2 ^b^	43.7 ± 8.1 ^b^

TS—Tensile strength, and E—Elongation at break. The measurement was performed in 10 repetitions. The parameters in the columns (value ± standard deviation) denoted with the same letters (a, b) do not differ statistically at the confidence level of *p* < 0.05.

**Table 3:** Water content, solubility, and degree of film swelling.

Sample	Water Content [%]	Solubility [%]	Degree of Swelling [%]
K	6.0 ± 1.1 ^a^	33.4 ± 0.5 ^a^	67.0± 3.7 ^a^
QD-ZnS	5.4 ± 1.5 ^a^	36.8 ± 0.7 ^b^	69.3 ± 0.8 ^a^
QD-CdS	5.2 ± 1.0 ^a^	34.1 ± 1.0 ^a^	66.2 ± 1.7 ^a^

The measurement was performed in 3 repetitions. The parameters in the columns (value ± standard deviation) with the same letters (a, b) do not differ statistically at the confidence level of *p* < 0.05.

**Table 4:** Water and diiodomethane wetting conditions.

Sample	Contact Angle		Surface Free Energy		
	Water	DIM	Dispersive	Polar	Total
K	69.7	37.8	39.32	7.65	46.97
QD-ZnS	76.2	34.5	43.24	4.10	47.32
QD-CdS	88.0	40.3	43.39	0.95	44.34

**Table 5:** Analysis of the sizes and zeta potentials.

Sample	Size [nm]	Zeta Potential [mV]
K	350	51.4
QD-ZnS	3600	−7.4
QD-CdS	510	35.6

## Data Availability

The data presented in this study are available on request from the corresponding author.
